# Biological autoluminescence as a noninvasive monitoring tool for chemical and physical modulation of oxidation in yeast cell culture

**DOI:** 10.1038/s41598-020-79668-2

**Published:** 2021-01-11

**Authors:** Martin Bereta, Michal Teplan, Djamel Eddine Chafai, Roman Radil, Michal Cifra

**Affiliations:** 1 Institute of Measurement Science of the Slovak Academy of Sciences, Bratislava, Slovakia; 2 Faculty of Health, Catholic University in Ruzomberok, Ruzomberok, Slovakia; 3 Institute of Photonics and Electronics of the Czech Academy of Sciences, Prague, Czechia; 4 Faculty of Electrical Engineering and Information Technology, University of Zilina, Zilina, Slovakia

**Keywords:** Bioenergetics, Photobiology, Imaging and sensing, Biological physics

## Abstract

Normal or excessive oxidative metabolism in organisms is essential in physiological and pathophysiological processes, respectively. Therefore, monitoring of biological oxidative processes induced by the chemical or physical stimuli is nowadays of extreme importance due to the environment overloaded with various physicochemical factors. Current techniques typically require the addition of chemical labels or light illumination, which perturb the samples to be analyzed. Moreover, the current techniques are very demanding in terms of sample preparation and equipment. To alleviate these limitations, we propose a label-free monitoring tool of oxidation based on biological autoluminescence (BAL). We demonstrate this tool on *Saccharomyces cerevisiae* cell culture. We showed that BAL can be used to monitor chemical perturbation of yeast due to Fenton reagents initiated oxidation—the BAL intensity changes with hydrogen peroxide concentration in a dose-dependent manner. Furthermore, we also showed that BAL reflects the effects of low-frequency magnetic field on the yeast cell culture, where we observed a disturbance of the BAL kinetics in the exposed vs. control case. Our results contribute to the development of novel techniques for label-free, real-time, noninvasive monitoring of oxidative processes and approaches for their modulation.

## Introduction

**Figure 1:**
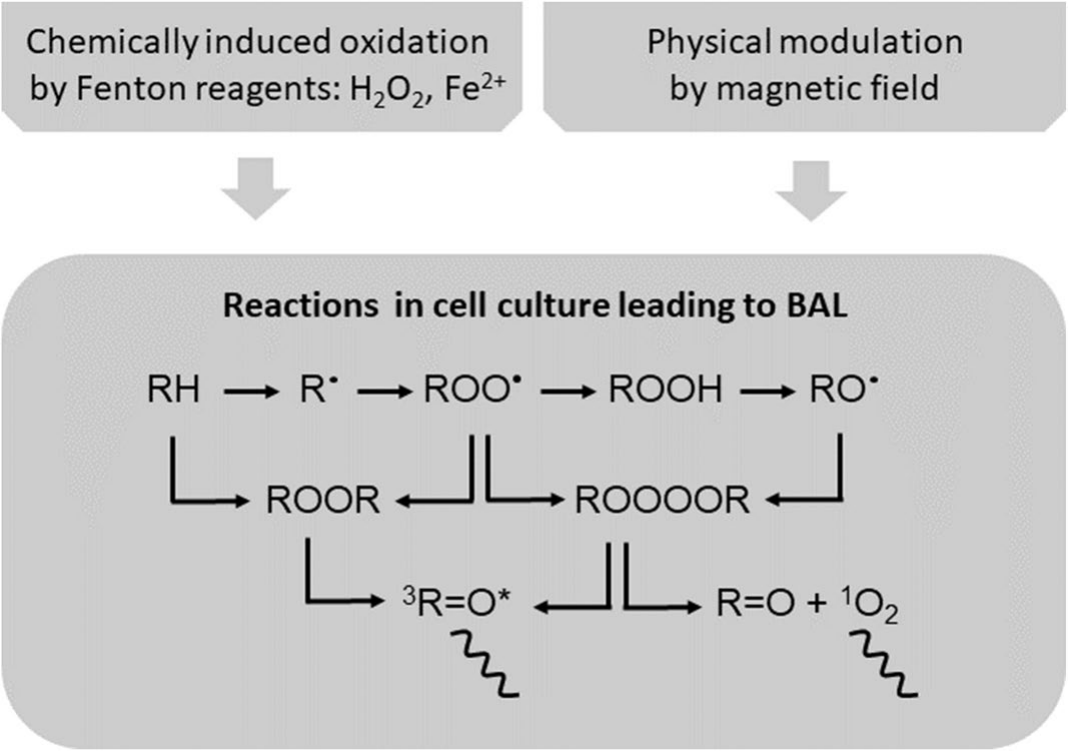
Schematic illustration on the mechanism of BAL under the chemical and physical modulation of yeast cells. The hydroxyl radical (either generated during endogenous metabolism or by addition of external precursors) causes oxidation of biomolecules (RH) and produces secondary radicals (alkyl radical $\hbox {R}^\cdot$, peroxyl radical $\hbox {ROO}^\cdot$, alkoxyl radical $\hbox {RO}^\cdot$ produced via nonradical biomolecular hydroperoxide ROOH). Those radicals lead to the formation of unstable intermediates dioxetane (ROOR) and tetraoxide (ROOOOR). These high-energy intermediates can be decomposed to electron-excited species, such as triplet excited carbonyl ($^3\hbox {R}=\hbox {O}^{*}$)^44–47^ or singlet oxygen ($^1\hbox {O}_2$)^48,49^. The transition of these species to the ground state is accompanied by the photon emission (wavy line) manifested as biological autoluminescence. BAL is also produced during cell culture cultivation when oxidative metabolic processes are present. BAL reaction scheme is adopted from^2^. In the case of chemically induced oxidation, hydroxyl radical is formed as a product of Fenton reaction and abstracts hydrogen from biomolecule RH to initiate the cascade of further reactions. The physical stimulus can affect the biological system at multiple points of the scheme: potentially affecting the rate of the endogenous oxidative metabolism or rate of the recombination of radical species.

The emission of light originating from biochemical processes in organisms is an externally detectable manifestation of the metabolic activity of living cells. There are several terms used for this kind of light emission, expressing different attributes of this phenomenon^1^. One of the most appropriate and concise terms is biological autoluminescence (BAL), which emphasizes both the biological origin and the endogenous generation of weak light emission. The biochemical reactions leading to BAL generation are initiated by reactive oxygen species (ROS)^1^. ROS oxidize biomolecules and these reactions can further lead to the formation of unstable intermediates, which decay to form electron-excited species^2^. The transition of these species to the ground state is accompanied by the photon emission in near UVA (350–400 nm), visible (400–750 nm), and near IR (750–1300 nm) regions of the electromagnetic spectrum (see reaction scheme in Fig. 1^1^). ROS themselves are produced during oxidative metabolic processes (*e.g.*, cellular respiration), but can also be formed as a product of stress-induced oxidative reactions^2^. BAL could be used for monitoring both physiological oxidative metabolism and also oxidative stress in organisms due to its noninvasive, low-operation-cost, and label-free application. Since oxidative stress is present in various diseases, the BAL can also be potentially employed in many fields of biomedicine^3,4^, such as dermatology^5^, neuroscience^6^, oncology^7^ or agricultural biotechnology^8–10^.

The modern environment exposes organisms to various new kinds of chemical and physical pollution which have not existed in the past centuries. Pollution may affect behavior^11^, cognitive functions^12^ and induce respiratory^13^, cardiovascular^14^ and cerebrovascular^15^ diseases. At the mechanistic level, many types of these pathophysiological endpoints are related to the oxidative stress at the cellular level^16,17^. Consequently, there is a great need to develop novel effective techniques to monitor chemical and physical factors which affect oxidative balance in organisms.

**Figure 2:**
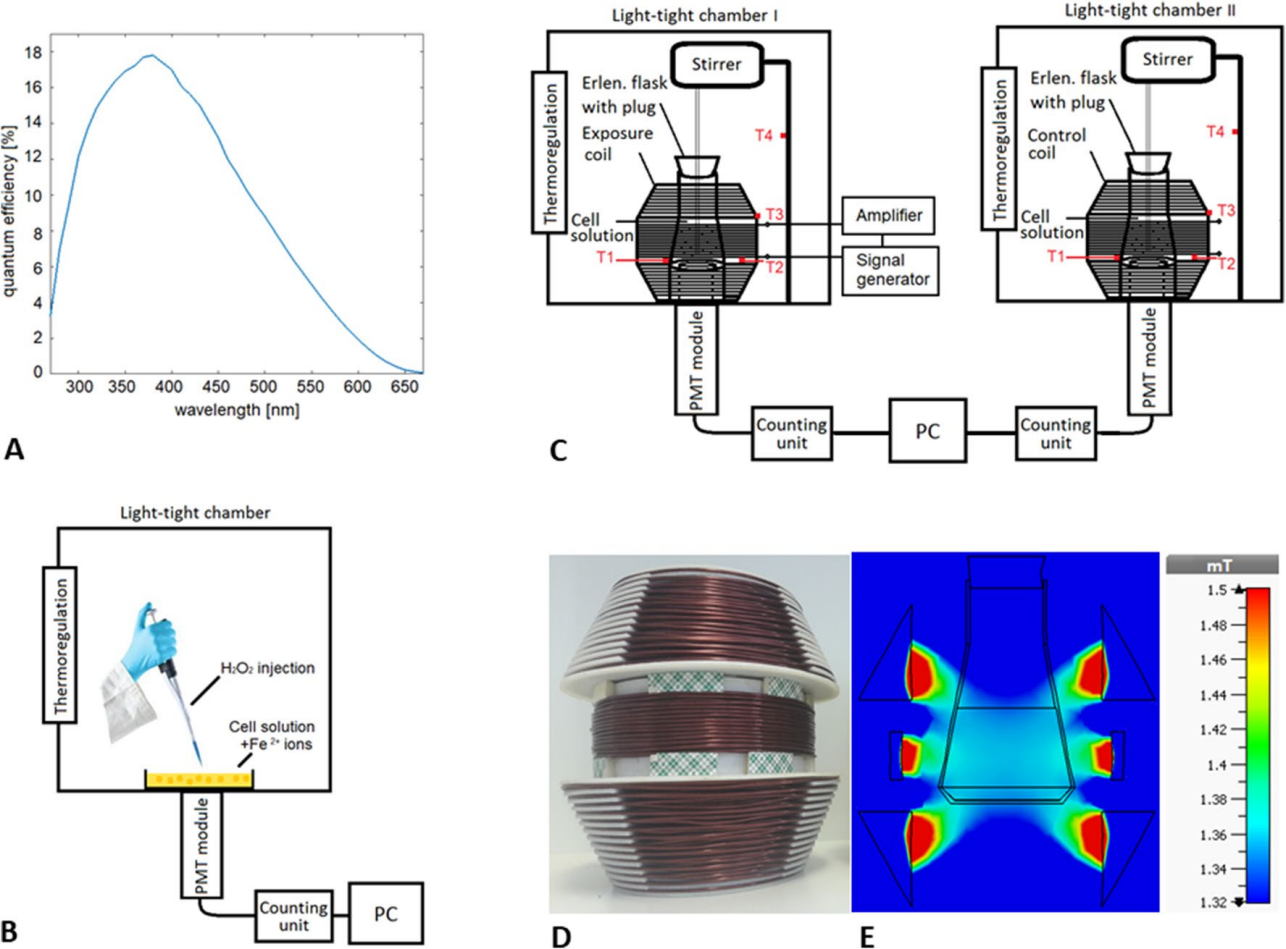
Methods for monitoring of chemical and physical modulation of yeast cell culture by using biological autoluminescence (BAL). (**A**) Quantum efficiency of the photomultiplier module used for BAL detection. (**B**) BAL monitoring during chemically induced oxidation of cell culture by Fenton reagents. (**C**) Light-tight chambers for monitoring of BAL ensure appropriate conditions for pair experiments. Two identical coils are used, one of them, labeled exposure coil, is connected to the signal generator to generate magnetic field. The second one, labeled control coil, is not connected to the generator and is used to ensure the same air flowing conditions in the chamber with the control sample. The placement of exposure coil with respect to the chambers was randomized. The samples are mechanically stirred to avoid sedimentation of cells. Temperature sensors (T1–T4) for recording the temperature during all experiments are placed at the same locations in both chambers in order to control the potential heating effect by exposure coil: T1 is attached to the Erlenmeyer flask, T2 is free-hanging in the coil cavity, T3 is attached to the outer side of the coil and T4 is attached to the stand of the stirrer to monitor the overall temperature in the chamber. (**D**) Exposure coil for magnetic field generation (photo). (**E**) Magnetic flux density distribution within the exposure area (volume of the cell culture) exhibits 95% homogeneity.

The chemical stimulus we exploit is the oxidation of cells via external addition of Fenton reagents (divalent iron ($\hbox {Fe}^{2+}$) and hydrogen peroxide ($\hbox {H}_2\hbox {O}_2$)) (Figs. 1, 2B). Iron is also naturally present in organisms^18^ and $\hbox {H}_2\hbox {O}_2$ is being continuously produced during oxidative metabolism in all aerobic organisms including yeast^19,20^. Therefore, Fenton reaction takes place also endogenously in organism. Fenton reagents generate hydroxyl radical ($\hbox {HO}^\cdot$), a highly reactive radical ROS^21,22^. $\hbox {HO}^\cdot$ can be generated in cells also endogenously^21^ and it has a very short lifetime due to its high reactivity. Iron-induced oxidation is a prominent group of oxidation treatments used both in biomedicine to model the effects of iron-excess diseases^18,23–26^ as well as in biotechnology in wastewater treatment^27^.

The physical stimulus we exploit, and which is widely discussed to be able to affect the cells and organisms is the magnetic field (MF). We hypothesize that the BAL dynamics could be used to monitor the effect of MF on organisms, under the assumption that MF affects the cellular metabolism (Figs. 1, 2C–E). The interest in the MF biological effects has increased due to the rapidly extending daily use of electronic devices and generally increasing the rate of human exposure to electromagnetic radiation from various artificial sources. Several studies indicated the potential connection between low-frequency (LF) MF exposure and cancer^28,29^ or cardiovascular diseases^30^. On the other hand, there are reports on the potential beneficial application of LF MF for the treatment of arrhythmia^31^ or polyneuropathy^32^. Numerous works focusing on experiments with living cells showed the impact of LF MF on the proliferation processes of cells or cell viability^33–37^. Besides experimental investigation, many authors proposed physical mechanisms of biological LF MF impact, such as ion cyclotron resonance^38^ and ion parametric resonance^39^ or radical pair mechanism^40–42^. However, although an extensive theoretical and experimental research has been carried out, the unambiguous explanation of LF MF influence on living structures is still lacking. Many recent findings in the area of biological effects of LF MF seem to be isolated, comparison between different reports is contradictory and inconclusive^43^. Hence, the research challenges for MF biological effects are still open, with potential novel applications in diagnostics, therapy as well as in industry. Since many open questions are not possible to be answered with current tools, it is essential to find novel methods for monitoring and evaluation of various facets of biological effects.

In this paper, we show the use of BAL as a tool to monitor the effects of selected chemical and physical stimuli and demonstrate the proof-of-principle on a standard model for eukaryotic cell biology: yeast *Saccharomyces cerevisiae*. We found that both chemical induction of oxidation via Fenton reagents and low-frequency magnetic field modulated oxidative metabolic reactions in yeast cell culture can be monitored by BAL.

## Results and discussion

### BAL for monitoring chemically induced oxidative stimulus

**Figure 3:**
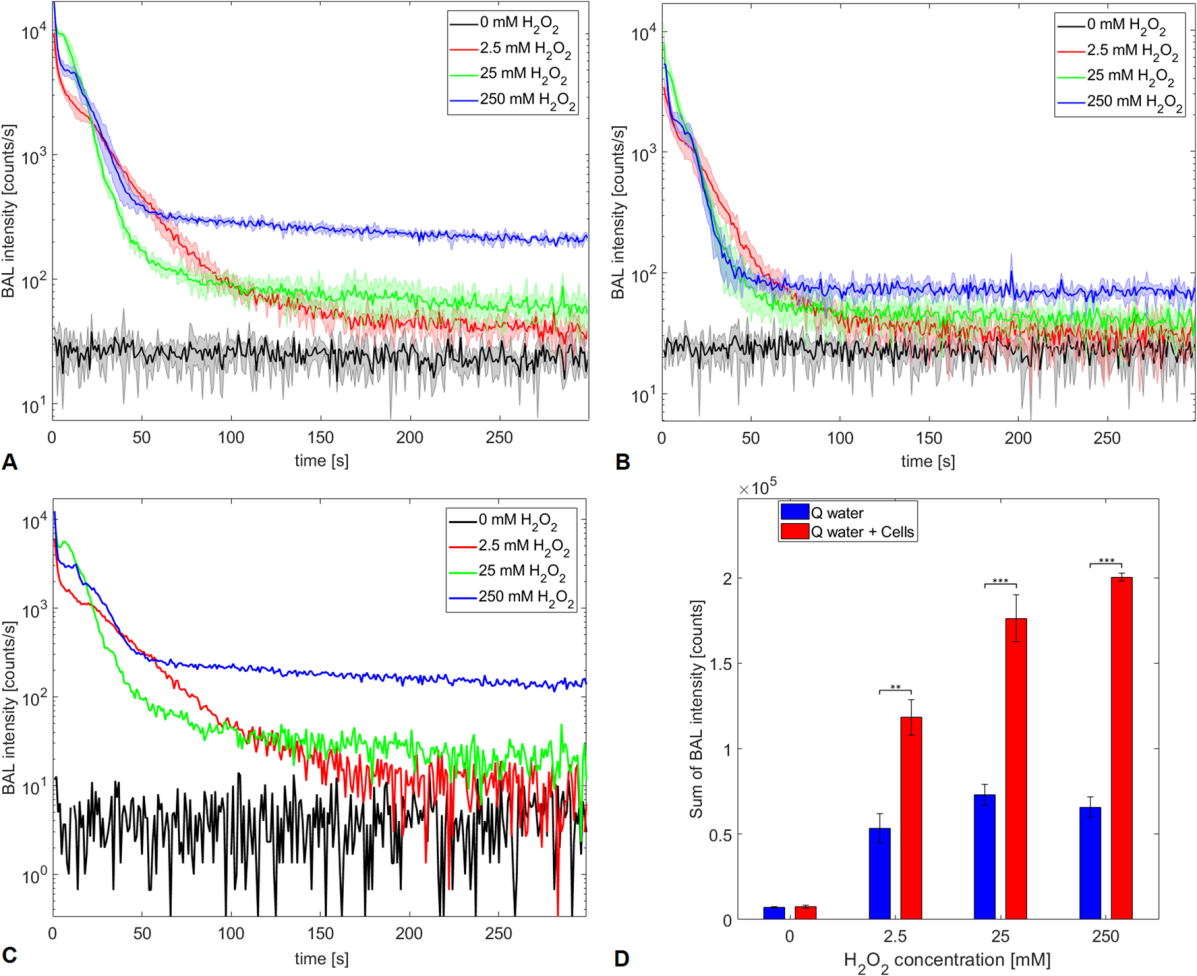
BAL from yeast cell culture under chemically induced oxidation. (**A**) The BAL time dynamics from yeast cell samples in purified water with three different concentrations of hydrogen peroxide $(\hbox {H}_2\hbox {O}_2)$ and fixed $\hbox {Fe}^{2+}$ concentration (0.5 mM). (**B**) The BAL time dynamics from control samples: purified water (Q water) with the same concentrations of hydrogen peroxide $(\hbox {H}_2\hbox {O}_2)$ and fixed $\hbox {Fe}^{2+}$ concentration. (**C**) The difference in BAL time dynamics between cell samples and control samples. The BAL signals from control samples (**B**) are subtracted from those with cells (**A**). (**D**) The sum of BAL intensity throughout the measurements, **$p<0.01$; ***$p<0.001$. The sum is calculated as the area under curves in **A**: Q water + Cells and (**B**) Q water. The data represent mean values from 3 repetitions of experiment and the error bars represent the standard deviation.

We show the potential use of BAL for monitoring chemically induced oxidation of yeast cells *Saccharomyces cerevisiae* (Fig. 3). The oxidation is initiated by highly reactive hydroxyl radical, which is formed in the solution from hydrogen peroxide via Fenton reaction. We aim to focus on the BAL that arises from the oxidation of yeast cell material, regardless if the cells are living or not. We present the results of BAL measurements from yeast cell samples with various concentrations of hydrogen peroxide, which is expected to produce various amounts of hydroxyl radical molecules in solution. Figure 3A shows the time dependence of BAL from yeast cell culture with the presence of ferrous ions after adding of three different concentrations of hydrogen peroxide $(\hbox {H}_2\hbox {O}_2)$. The unit counts per second (counts/s) represents the number of photons detected by a photomultiplier module in 1 s. The length of each measurement is 300 s.

The initial maximum of BAL kinetics (Fig. 3A) is likely caused by fast oxidation of yeast cells by hydroxyl radical created immediately after hydrogen peroxide application. This response of BAL can be conceptually explained by the Fenton reaction kinetics models in biomolecular solutions^50,51^. The decreasing BAL kinetics in all the concentrations probably reflects the progressive consumption of secondary radicals produced in oxidative reactions leading to non-reactive products formation. The higher the hydrogen peroxide concentration, the higher BAL intensity throughout the measurements (Fig. 3A) is observed.

We additionally induce Fenton reaction in purified water (Fig. 3B), in order to detect BAL response to this chemical process in the water without cells. The similar trend of BAL signals as in the case of cells is observed, but the signal levels are lower, as can be seen in subtracted signals (Fig. 3C). Ivanova et al.^51^ propose mechanisms, where Fenton reagents in water generate singlet oxygen via a series of reactions. Singlet oxygen then emits light either via monomol or dimol emission manifesting as BAL.

The significantly higher total BAL counts (Fig. 3D) calculated as the area under the curves in Fig. 3A, compared to the control case (Fig. 3B) are obtained. With the increasing concentration of hydrogen peroxide applied to the solution, the increasing amount of hydroxyl radical molecules in Fenton reaction is produced. The total amount of electron-excited species responsible for BAL generation is then very likely increased and therefore higher BAL intensity is detected.

To summarize, we propose BAL as a useful and innovative non-invasive method for monitoring of cell culture oxidation. However, in order to develop a reliable assessing method, further studies are necessary to conduct.

**Figure 4:**
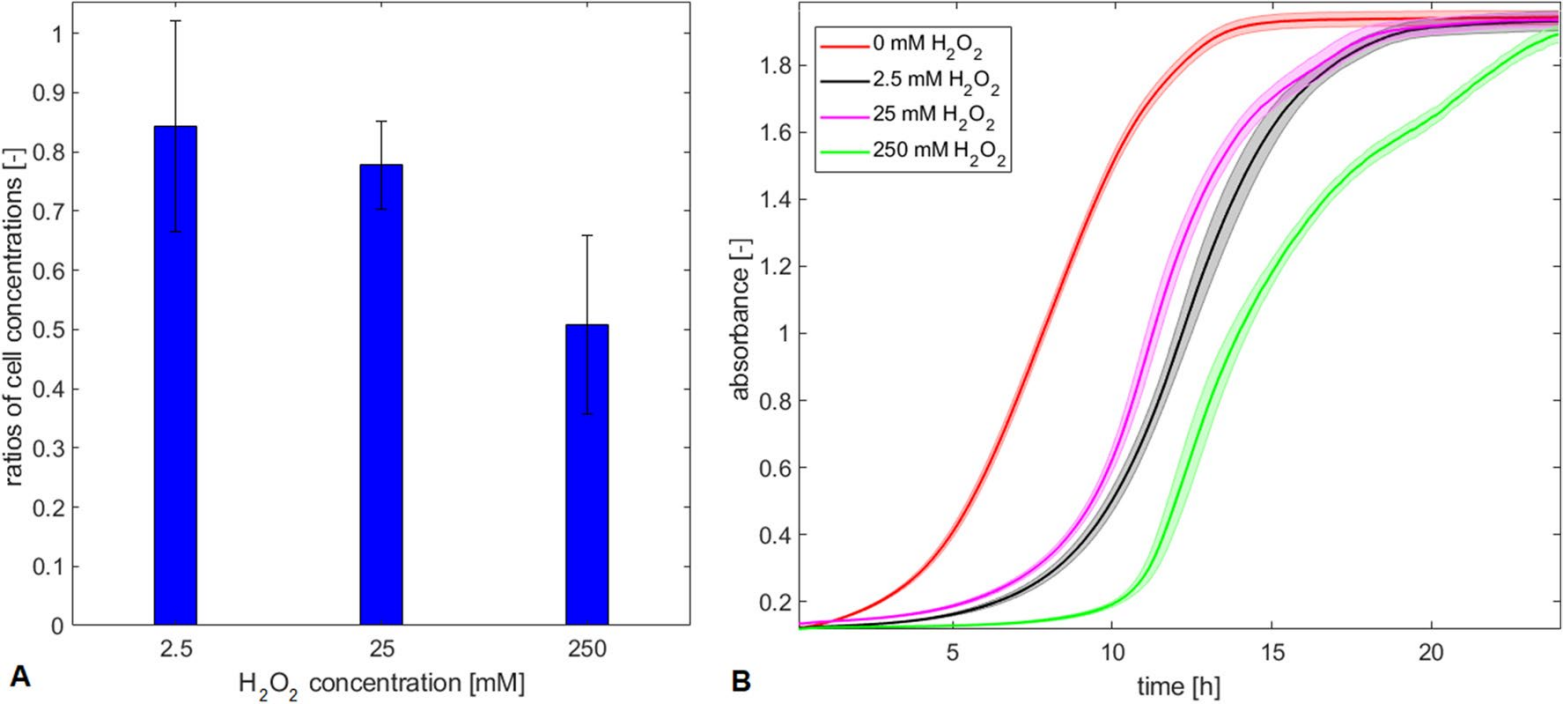
Cell concentration and cell culture growth after chemically induced oxidation treatment. (**A**) Ratios of cell concentration in the oxidized sample to concentration in the control sample after oxidation treatment. (**B**) Cell culture growth after oxidation treatment. The data represent mean values from $\hbox {n}=4$ repetitions (**A**) and from $\hbox {n}=12$ repetitions (**B**) of each experiment and the error bars constitute the standard deviation.

**Figure 5:**
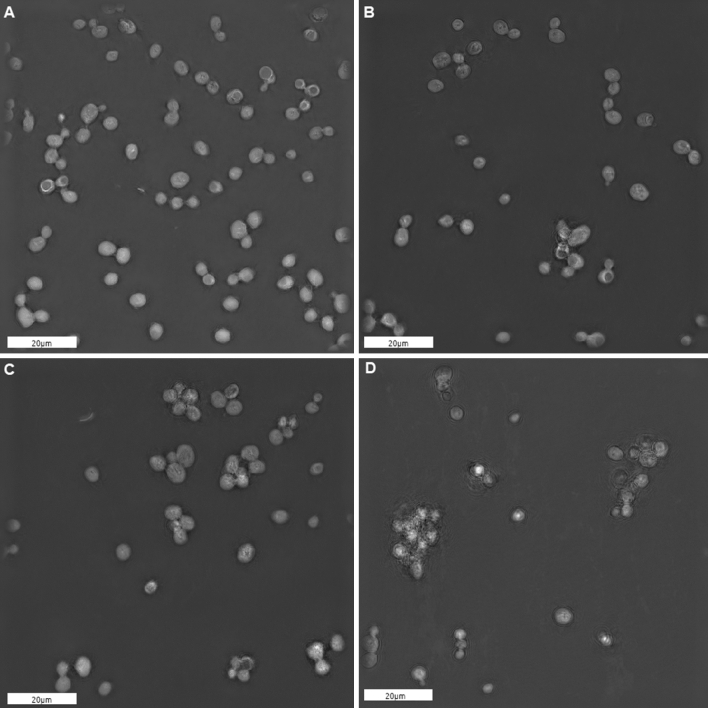
Microscopy of yeast cells after chemically induced oxidation treatment. Three different concentrations of hydrogen peroxide $(\hbox {H}_2\hbox {O}_2)$ and fixed $\hbox {Fe}^{2+}$ concentration (0.5 mM) were applied. White bars are 20 $\mu \hbox {m}$ long. (**A**) Image of control (untreated) cell sample (0 mM $\hbox {H}_2\hbox {O}_2$). (**B**) Image of oxidized cell sample (2.5 mM $\hbox {H}_2\hbox {O}_2$). (**C** Image of oxidized cell sample (25 mM $\hbox {H}_2\hbox {O}_2$). (**D**) Image of oxidized cell sample (250 mM $\hbox {H}_2\hbox {O}_2$).

### BAL signal relationship to physiostructural damage of cells

To deeper analyze the increase of BAL signal with increasing $\hbox {H}_2\hbox {O}_2$ concentration and its relationship to oxidation effects on cell culture, we performed a size exclusion assay. In this assay, we measure the cell concentration and size of untreated cells. The concentration of treated cells was measured, but taking into account that only those cells that fall in the diameter range of untreated cells are considered. The concentration of cells was measured in the range of untreated cells 3-9 $\mu \hbox {m}$, which is the typical range for the diameter of a single yeast cell. The results are evaluated as the ratios of cell concentrations in treated and untreated samples. It can be seen that the ratios are decreasing with increasing $\hbox {H}_2\hbox {O}_2$ concentration (Fig. 4A), although not significantly. We assume that the higher the concentration of hydrogen peroxide applied to the solution, the more cells are excluded, and therefore the lower concentration in the selected diameter range (3-9 $\mu \hbox {m}$) was measured. This is probably due to the following reasons: either the cells swelled, clustered, or fragmented. We also performed a cell viability test after oxidation using trypan blue staining. The viability decreases to 87.7% in case of oxidation by 250 mM of $\hbox {H}_2\hbox {O}_2$, while for 25 mM, 2.5 mM, and control case, viability exceeded 98.4%.

To analyze cell activity after oxidation, we additionally monitored cell growth after oxidation (Fig. 4B) in multiwell plate reader (Tecan Spark). The results indicate the latency in onset of the exponential phase of cell growth curve as well as in reaching saturation (stationary phase) when oxidation is induced, compared to no oxidation treatment (0 mM $\hbox {H}_2\hbox {O}_2$). The latency tends to increase with the increasing $\hbox {H}_2\hbox {O}_2$ concentration with 2.5 and 25 mM conditions giving results similar to each other. The seemingly reverse mutual order of latency might be explained by the fact, that the initial concentrations of cells, which were able to grow and divide, were also in seemingly reverse order for these two particular samples (2.5 and 25 mM). It means the initial concentration of cells able to grow and divide in a sample which underwent oxidation by 25 mM of $\hbox {H}_2\hbox {O}_2$ was lower than that one of 2.5 mM. Therefore we observed reverse mutual order of latency in onset of the exponential phase of growth curves. However, the results of the identical experiment^52^ yielded the expected order of the latency in the growth curves.

This observation can be linked to the size exclusion test results presented in Fig. 4A. The lowest concentration of cells was detected after the strongest oxidation treatment (Fig. 4A, 250 mM $\hbox {H}_2\hbox {O}_2$). Similarly, the latest onset of the exponential phase was observed in this condition (Fig. 4B). At this point, we hypothesized that the cells, which underwent physiostructural damage, either swelling, clustering, or fragmentation, were not able to grow and divide.

To further understand cell behavior under the oxidation treatment, we performed 3D holotomographic microscopy to image our cells (Fig. 5, Fig. S1). It is clearly shown that untreated cells are intact and well suspended (Fig. 5A). Contrary to this control, treated cells tend to form clusters (Fig. 5B–D), moreover, the refractive index of the suspension was slightly changed (data not shown). Finally, based on our observations, we can assume that increasing concentration of $\hbox {H}_2\hbox {O}_2$ leads to stronger oxidation causing an increasing rate of physiostructural damage (Fig. 5B–D), which finally results in increasing of BAL levels (Fig. 3).

**Figure 6:**
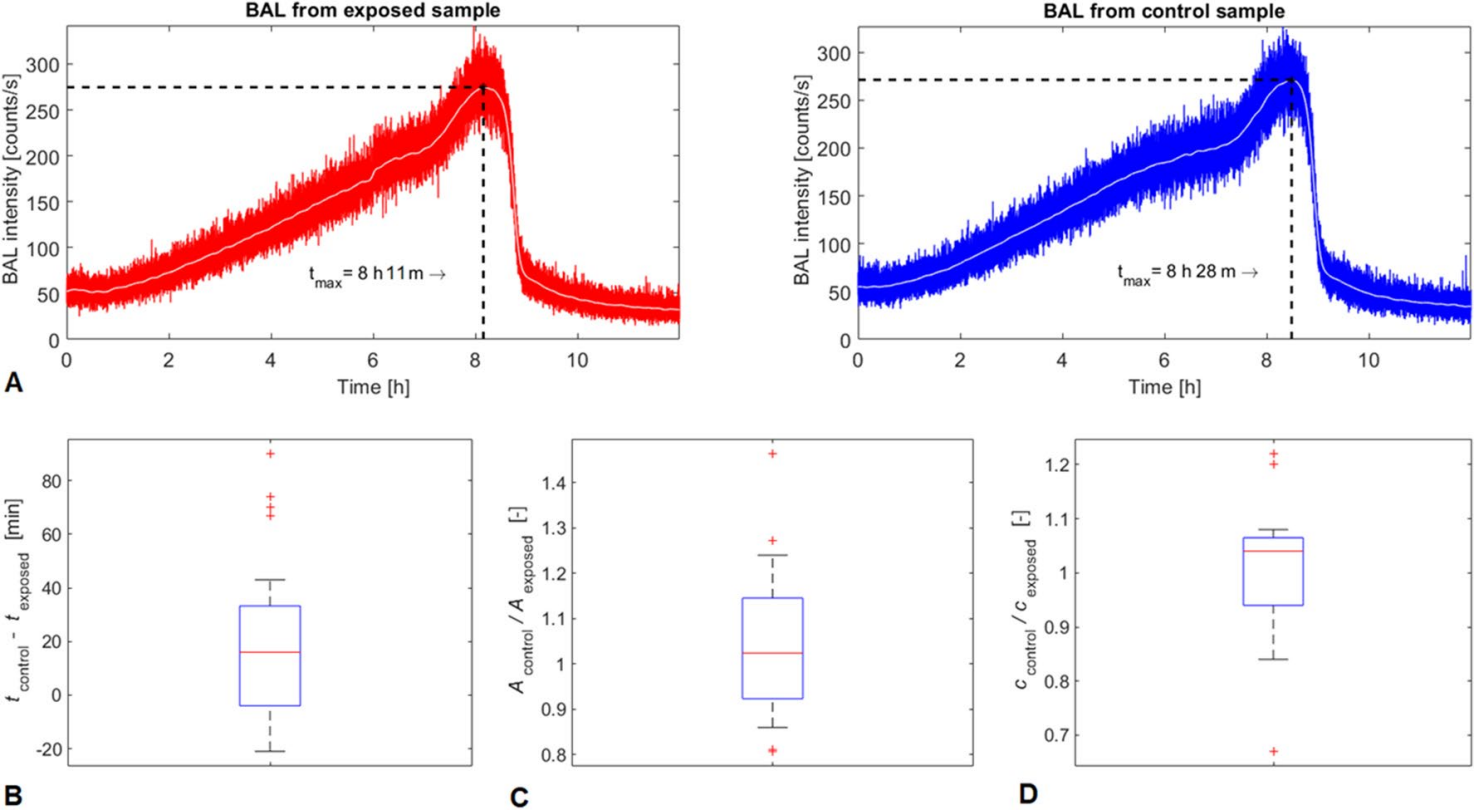
BAL monitoring and cell concentration in yeast cell culture exposed to low-frequency magnetic field. (**A**) The representative shape of BAL dynamics from magnetic field exposed and control sample of yeast cells. (**B**) Boxplot of time differences of BAL maxima reaching between exposed and control sample (*t*_control_ − *t*_exposed_) shows that the maximum tends to occur earlier in exposed samples ($p=0.039$). (**C**) Boxplot of ratios of BAL maxima intensities (*A*_control_/*A*_exposed_). (**D**) Boxplot of ratios of cell concentrations measured at the time of 6 h (*c*_control_/*c*_exposed_).

### BAL for monitoring of physically modulated yeast cells’ oxidative metabolism by magnetic field

Since metabolic processes in cells involve oxidative reactions, shown previously to be detectable by BAL^53^ and LF MF was reported to be able to affect yeast *Saccharomyces cerevisae* cells growth dynamics^37,54,55^, we aim to examine, whether BAL dynamics can reflect LF MF effects on cells. Following our previous results which indicate a proliferative response of cells to LF MF in the frequency range 1-2 kHz^56^ we chose for this study similar exposure parameters (800 Hz,  1.5 mT) and we monitor BAL throughout the exposure of yeast cells to LF MF. For long-term BAL measurement (12 h) during our MF experiments, similar BAL dynamics for exposed as well as for control sample is typically observed (Fig. 6A). The distinct maximum of BAL intensity is typical for every measurement (Fig. 6A). Additionally, the fast decrease of BAL intensity is always obtained after reaching this maximum (Fig. 6A). Our results are corroborated by earlier data by Quickenden and Tilbury^57–61^ where dynamics of increasing BAL with a sudden drop at certain time point was observed during the growth of the yeast cell culture. In general, since the dynamics of BAL is related to metabolic processes of cells, the specific characteristics of this dynamics could be the result of biochemical shifts during cell metabolism.

Therefore, from paired control-MF experiments (n = 21) we have decided to evaluate time instants when the intensity of BAL reaches the mentioned maximum. The results in a form of the time differences in reaching BAL maxima between control and exposed sample (Fig. 6B, Tab. S1) indicate that the maximum tends to occur earlier in exposed samples ($p=0.039$). The ratios of BAL maxima intensities (Fig. 6C, Tab. S1) do not indicate any significant differences between the exposed and control samples.

Since there are a number of studies^37,54–56,62,63^ investigating the magnetic field effect on growth dynamics of yeast cells, we also hypothesized, the magnetic field in our experimental conditions could affect cell growth rate. Therefore, we decided to measure the cell concentration at a specific time point (6 h after the start of each experiment) when cultivated cell cultures are in the exponential phase of the growth cycle. The results, in this case, do not indicate any significant differences between exposed and control samples (Fig. 6D, Tab. S1). It seems that although the magnetic field in these experimental conditions does not have any observable influence on the cell concentration, it could alter the biochemical reactions during the cell metabolism since the time shift in reaching BAL maxima is observable. To obtain an insight into the biochemical background, we also realized a single measurement of dissolved oxygen concentration in the yeast cell sample simultaneously with BAL (Fig. S2). This measurement reflects the rate of oxygen consumption during cell metabolism and population growth. It can be seen that a mutual relation between the BAL and oxygen level is rather complex. However, especially from the final extreme drop of both BAL and oxygen level we can assume that, at least during certain circumstances, the BAL kinetics reflects the oxidative metabolism of cell culture. This is supported by the investigation of Quickenden and Tilbury^59^, where the absence of oxygen eliminates BAL.

Since BAL kinetics is tightly connected to the growth of the cell culture, we suggest that there is a slight MF influence on yeast metabolism, not observable by cell concentration measurement, but detectable by using BAL. This could indicate beneficial use of BAL for monitoring of weak effects of MF on cells, which are not detectable by standard techniques of cell culture state characterization. Moreover, it may also contribute to understanding the inconsistency and irreproducibility of experimental results due to often hardly measurable LF MF biological effects. However, further studies are needed to better understand the processes behind the disturbance of the BAL curve.

### Limitations and future work

Does the BAL originate in the intracellular or extracellular space? From the current data, it is impossible to disentangle the exact location of BAL emitters, i.e. the location where molecules in the electron excited state emit the photon from. Considering the general scheme in Fig. 1, this question has several levels. At first, the question is reasonable, only when we argue that the presence of cells is inevitable for BAL generation. In general, we believe that the reactions, which generate primary ROS that lead to BAL are taking place only when cells are present in the medium. The secondary ROS can be produced in the cells or outside the cells, hence final electron excited state molecules leading to BAL can be produced in the cells or outside the cells. In short, we believe that without active cell oxidative metabolism (which is definitely present during long-term cell cultivation in YPD medium) there is no ROS formation and following BAL response. There are several experimental observations which support these claims. At first, we know that the absence of cells does not lead to the typical BAL curve with slow increase and sharp decrease, see Supplementary data of the Vahalová et al.^52^. At second, after the BAL signal drops, the addition of glucose (primary carbon source for yeast under given conditions) leads to a partial recovery of the BAL intensity, indicating that cellular metabolism is involved (see again Supplementary data of the Vahalová et al.^52^). Furthermore, we showed in our earlier work^64^ that addition of antioxidants (particularly ascorbic acid) decreases BAL signal. Finally, evidence pointing out towards the intracellular origin of BAL comes from images of yeast cell clusters spread on agar medium^65^. In this case, a solid growth medium in a form of YPD agar is chemically similar to our liquid YPD medium, meaning that a potential for extracellular BAL generation is somewhat similar. The only difference is in diffusion properties of each medium.

In order to address this limitation regarding the spatial origin of the BAL, we propose the procedure that could discriminate whether the BAL origin comes mainly from the cells or from the media solution. For this task, one can exploit a microscopic or macroscopic approach. The former is based on analysis of spatial BAL picture obtained from a sensitive camera, where individual cells or their clusters emit excessive BAL. However, it is close to impossible to detect the image of the BAL signals from individual cells because of the low photon fluxes^65^. On the other hand, the latter approach requires either to treat the cells chemically or to separate them, e.g. by filtration through the semi-permeable membrane or by other physical processes. It seems that the exploitation of sedimentation could be the right experiment for a decision regarding the spatial origin of BAL.

## Conclusion

In the study, we demonstrated a label-free monitoring of chemical and physical stimuli on yeast cells using BAL. The BAL intensity from yeast cell culture under the chemically induced oxidation is dependent on the hydrogen peroxide concentration applied to the solution. The observed results showed the key role of Fenton reaction, likely due to its hydroxyl radical product, in oxidation processes leading to the BAL from yeast cell culture. Although the exact mechanism of oxidative stress-induced BAL from yeast cell culture is not currently known, the presented results are consistent with theoretical assumptions suggesting the reactive oxygen species as the initiators of reaction pathways leading to BAL origin. Moreover, we showed the physiostructural damage of cells after oxidation by measurement of cell concentration, cell viability, cell culture growth, and microscopy imaging as well.

Furthermore, we detected a disturbance of BAL kinetics from yeast cell culture under the influence of low-frequency magnetic field, whereas cell concentration measurement did not indicate any significant difference between magnetic field exposed and control sample. This finding indicates the potential use of BAL for the detection of weak effects of magnetic field on cells, which is not observable by standard techniques of cell culture characterization. The obtained results contribute to the development of innovative approaches for label-free, real-time, noninvasive monitoring of oxidative processes and procedures for their modulation.

## Materials and methods

### Yeast culture growth conditions

The yeast culture *Saccharomyces cerevisiae* (genetic background BY4741, MATa) used in the experiments is stored on agar plates (1% (w/v) yeast extract, 2% (w/v) peptone, 2% (w/v) agar, 2% (w/v) D-glucose in purified water) in a refrigerator at $4^\circ \hbox {C}$. Yeasts are inoculated from agar plate into glass Erlenmeyer flask (250 mL) with 100 ml of YPD medium (1% (w/v) yeast extract, 2% (w/v) peptone, 2% (w/v) D-glucose in purified water) and cultivated for 24 h at $30^\circ$ C on an orbital shaker (Biocer) at 180 rpm.

### Biological autoluminescence measurement setups

The photomultiplier (PMT) module H7360-01, selected type (Hamamatsu Photonics K.K.) with a spectral sensitivity in the range of 300-650 nm is used to detect biological autoluminescence. The quantum efficiency of the PMT module is displayed in Fig. 2A. Typical dark count (noise) of PMT module H7360-01 is about 15 counts per second. The measurement of the sample takes place in a light-tight chamber (standard black box, Institute of Photonics and Electronics, CZ) specially designed for the purposes of BAL measurements. The PMT module is mounted on the bottom of the chamber, viewing the sample inside the chamber. For monitoring of BAL from yeast cell culture under the chemical stimulus, we performed single-chamber measurements (Fig. 2B). For monitoring the physical stimulation of cells, pair measurements of BAL in two identical chambers were carried out (Fig. 2C).

### Oxidation treatment conditions for BAL monitoring experiments

The precultivated cell culture is centrifuged twice at 3000 rpm, each time for 5 min and washed with purified water (Millipore Mili-Q). Cell concentration is measured by cell counter (Beckmann Coulter) and the 50 mL stock solution is set to a concentration of $1\times 10^{8}$ cells/mL. The chemical compounds used for oxidation are 30% hydrogen peroxide (Penta, CZ, p.a.) and ferrous sulphate heptahydrate (Penta, CZ, purity 99%). The sample of yeast cell culture from stock solution (3 mL, $1\times 10^{8}$ cells/mL) is transferred on Petri dish (diameter 35 mm) and ferrous sulphate heptahydrate ($\hbox {FeSO}_{4}\times 7\hbox {H}_{2}\hbox {O}$) is added. The final concentration of iron is 0.5 mM in each experiment. The sample is placed into the light-tight chamber and BAL measurement is started. Then the hydrogen peroxide ($\hbox {H}_{2}\hbox {O}_{2}$) is added to initiate Fenton reaction. Three different final concentrations of hydrogen peroxide (2.5 mM, 25 mM, and 250 mM) are used for experiments. BAL measurement for each hydrogen peroxide concentration is performed in triplicate.

### Cell concentration, cell size exclusion test, cell viability, cell growth measurement, and microscopy after oxidation treatment

The precultivated cell culture is centrifuged twice at 3000 rpm, each time for 5 min and washed with purified water. Cell concentration is measured by cell counter (Beckmann Coulter) and diluted in 200 mL stock solution (purified water) to concentration $1\times 10^{8}$ cells/mL. The sample of yeast cell culture from stock solution (30 mL) is transferred to Erlenmeyer flask (50 mL) and the same final concentrations of Fenton reagents ($\hbox {FeSO}_{4}\times 7\hbox {H}_{2}\hbox {O}, \hbox {H}_{2}\hbox {O}_{2}$) are applied to the solution, as in the BAL monitoring experiments. The samples are oxidized for 15 min at $30^\circ$ C on an orbital shaker (Biocer) at 180 rpm. Then the sample is centrifuged twice at 3000 rpm, each time for 5 min, washed with purified water, and stirred at the vortex. For the cell exclusion test, the cell concentration after oxidation in each sample is measured by the cell counter (Beckmann Coulter). The cell size range to be detected by the cell counter is set to 3–9 $\mu \hbox {m}$. For the cell viability test after oxidation, we used trypan blue solution (0.4%), stained cells for 5 min and counted the number of viable cells on Burker chamber by using optical microscope (Olympus BX 50). To monitor cell growth, we use Multiwell Plate Reader (Tecan Spark) and measure absorbance in 96-well plate at 600 nm for 24 h at $30^\circ$ C. The measurement time step was 10 min. The cell samples after oxidation treatment are diluted into 12 wells for each concentration of $\hbox {H}_{2}\hbox {O}_{2}$. The absorbance from untreated samples with the same initial concentration and from pure YPD medium is also measured as control samples. For microscope images capturing of cells treated by oxidation, cell culture is diluted to concentration $5\times 10^{6}$ cells/mL. The microscopy images of oxidized cell sample are captured on Holotomographic Microscope (The 3D cell explorer, Nanolive) to analyze physiostructural damage of cells.

### Magnetic field treatment conditions for BAL monitoring experiments

The cell concentration of precultivated cell culture is measured by the cell counter (Beckmann Coulter). The solution of cell culture is then diluted into two Erlenmeyer flasks (250 mL) containing 150 mL of liquid cultivation medium YPD to set the same initial concentration ($5\times 10^6$ cells/mL) in both samples. Pair measurements of BAL in two identical light-tight chambers are performed. One sample is exposed to magnetic field and one sample is nonexposed (control sample). Magnetic field is generated by the coil (Fig. 2D), designed previously^66^ to achieve 95% homogeneity in the exposure area (Fig. 2E). The coil is fed by a harmonic driving signal with frequency 800 Hz, generated by a signal generator (Agilent E4436B, Agilent Technologies, Inc.) and amplified by a linear amplifier (Hubert A1110-05, Dr. Hubert GmbH). Simulation of magnetic and electric field distribution within the coil for our experimental conditions (frequency 800 Hz, amplitude 1 A) was performed in CST Studio Suite 2018 (Fig. 2E, Fig. S3). In the simulation, the conductivity of cell solution was set to $\sigma =1$ S/m and relative permittivity $\epsilon =80$. The simulation was experimentally verified, the magnitude of magnetic flux density varied between 1.50 and 1.54 mT in the exposure area (measured by Gauss/Tesla meter 7010, F.W.Bell). Considering the noise of gaussmeter during measurement was ca. 0.08 mT, values from the measurement and simulation (Fig. 2E) match reasonably well. The schematics of exposure setup for pair measurements can be seen in the Fig. 2C. BAL from both samples is measured for 12 h. The samples are cultivated at $\hbox {30}^\circ$ C and are mechanically stirred to avoid sedimentation of cells. The temperature in 4 positions in each chamber (Fig. 2C) is recorded during each measurement by two identical 4-channel thermometers (Voltcraft PL-125-T4).

### Data analysis

BAL raw data were preprocessed and smoothed using Matlab, version R2019b (MathWorks, Inc.). In the case of magnetic field experiments, BAL curve characteristics were analyzed. Two particular features were extracted from the curve shapes: (1) the time point when BAL intensity reached its maximum, (2) the value of BAL intensity at this time point. For statistical analysis, only those measurements were selected, which passed through the following criteria: (1) temperature difference $\Delta$ $\hbox {T}<\hbox {0.5}^\circ$ C for mean values between chamber with the exposed and chamber with the control sample, calculated from all four temperature channels (Fig. 2C); (2) BAL curve shape without distortions caused by biological (contamination) and technical (stirring adjustment) issues during experiments. For the statistical analysis, unpaired two-sample t-test was performed for the datasets when normal distribution of data was not refused (BAL and concentration of cells under oxidation treatment) and paired Wilcoxon signed-rank test was performed for the cases when normal distribution of data was refused (BAL from cells under magnetic field treatment).

## Supplementary Information

Supplementary Information

## Data Availability

The datasets generated and analysed during the current study are available from the corresponding author on reasonable request.
