# Supplementary material for: Biological autoluminescence as a noninvasive monitoring tool for chemical and physical modulation of oxidation in yeast cell culture
Source: Sci Rep. 2021 Jan 11;11:328. doi: 10.1038/s41598-020-79668-2 (PMC7801494; doi:10.1038/s41598-020-79668-2)
Supplement: Supplementary file 1 — Supplementary Information [file 41598_2020_79668_MOESM1_ESM.pdf]

Supplementary information for: ”Biological autoluminescence as a noninvasive monitoring tool for chemical and physical modulation of oxidation in yeast cell culture”

Martin Bereta[1,2], Michal Teplan[1], Djamel Eddine Chafai[3], Roman Radil[4], and Michal Cifra[3,*]

[1]Institute of Measurement Science of the Slovak Academy of Sciences, Bratislava, Slovakia
[2]Faculty of Health, Catholic University in Ruzomberok, Slovakia
[3]Institute of Photonics and Electronics of the Czech Academy of Sciences, Prague, Czechia
[4]Faculty of Electrical Engineering and Information Technology, University of Zilina, Slovakia
[*]cifra@ufe.cz

ABSTRACT

This is the supplementary information containing additional data in form of figures and a table

Content

1. The 3D holotomographic microscopy reconstruction of yeast cells after chemically induced oxidation treatment: Fig. S1

2. The complete set of characteristics of BAL dynamics and cell concentration values from low frequency magnetic field experiments: Tab. S1.

3. BAL time evolution from yeast cell culture with the data of dissolved oxygen concentration: Fig. S2.

4. Figures of exposure coil model and simulation results of magnetic flux density and current density distribution: Fig. S3.

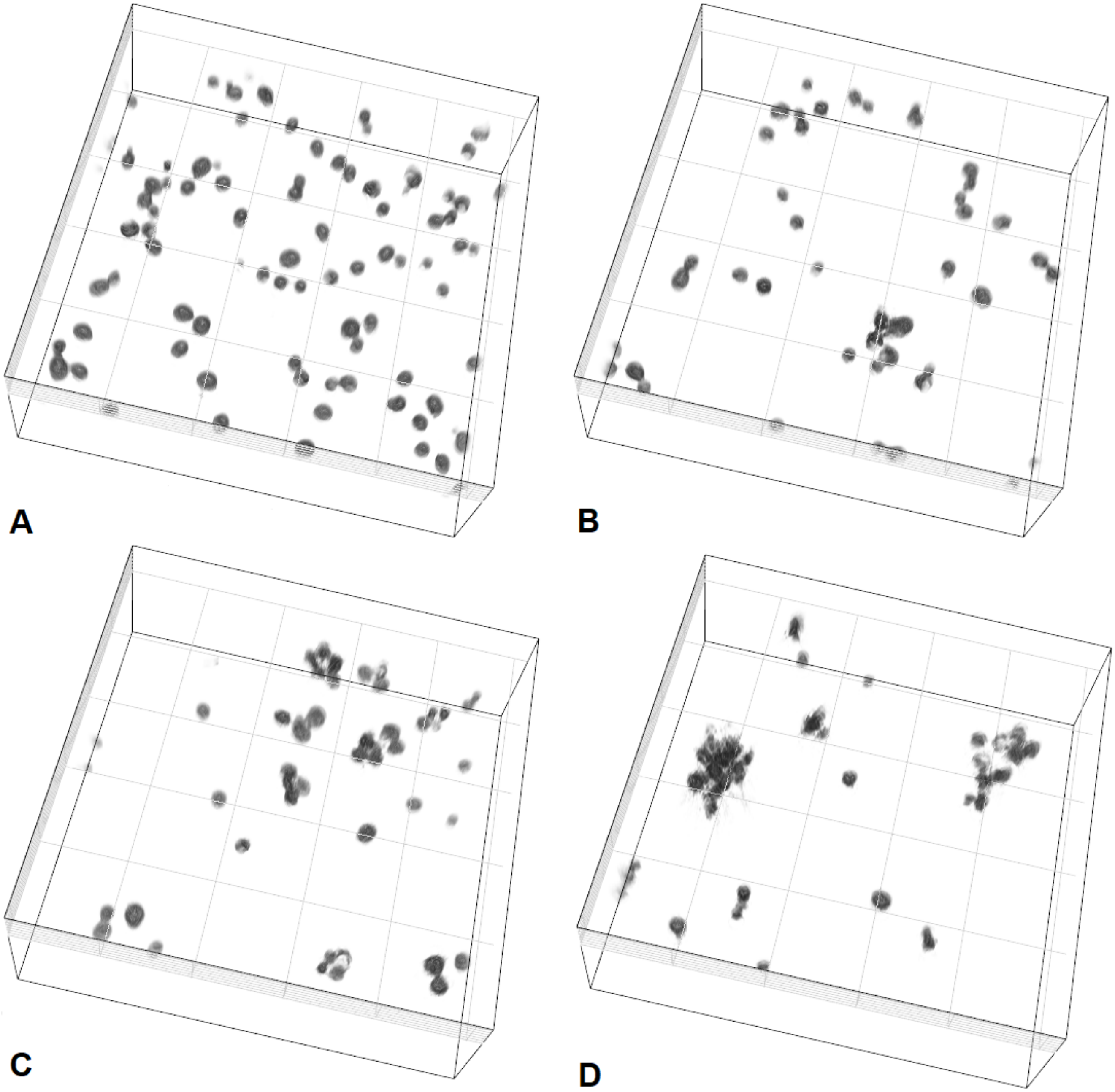

Figure S1. Nanolive holotomography microscopy image of yeast cells after chemically induced oxidation treatment. Three different concentrations of hydrogen peroxide (H_2O_2) and fixed Fe^{2+} concentration (0.5 mM) were applied. **A** Image of control cell sample. **B** Image of oxidized cell sample (2.5 mM H_2O_2). **C** Image of oxidized cell sample (25 mM H_2O_2). **D** Image of oxidized cell sample (250 mM H_2O_2).

Table S1. The complete set of characteristics of BAL dynamics and cell concentration values from low frequency magnetic field experiments: the time differences of BAL maxima reaching between exposed and control sample ($t_{control}$-$t_{exposed}$); the ratios of BAL maxima intensities ($A_{control}/A_{exposed}$); the ratios of cell concentrations ($c_{control}/c_{exposed}$) measured at the time of 6 hours.

No.	$t_{control}$ - $t_{exposed}$ [min]	$A_{control}$ / $A_{exposed}$ [-]	$c_{control}$/$c_{exposed}$ [-]
1	17	1.20	1.05
2	-4	0.91	N/A
3	90	1.07	N/A
4	5	1.11	N/A
5	43	1.01	N/A
6	16	1.13	0.84
7	17	0.98	1.08
8	1	0.86	0.93
9	67	1.27	1.06
10	-8	0.81	1.04
11	-4	1.11	0.93
12	16	1.01	1.20
13	30	1.46	1.07
14	-10	0.88	1.04
15	4	0.95	0.67
16	70	1.24	N/A
17	5	1.02	1.04
18	22	1.07	1.05
19	74	1.23	1.00
20	-21	0.81	0.95
21	-16	0.93	1.22

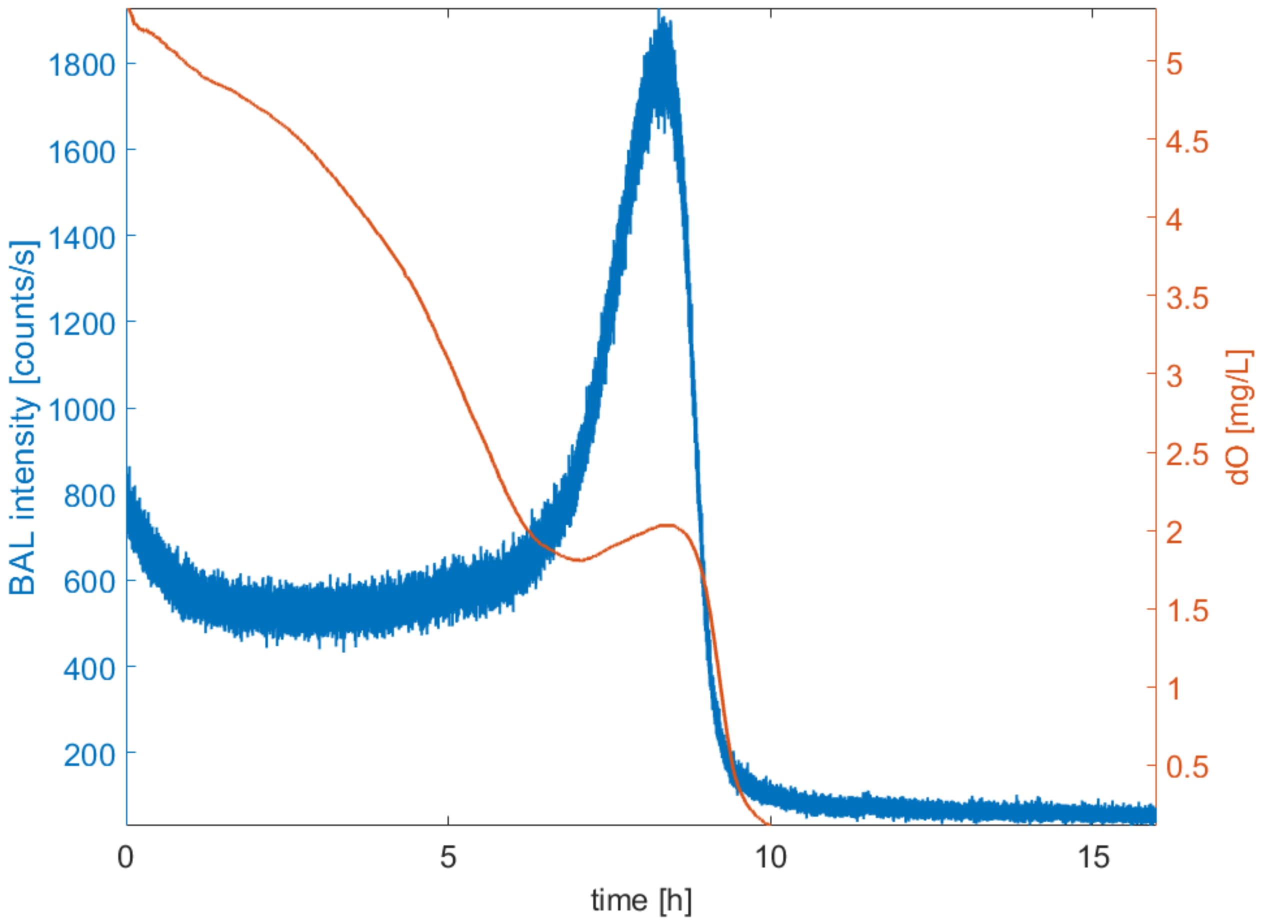

Figure S2. Time evolution of BAL from growing yeast cell culture together with dissolved oxygen concentration.

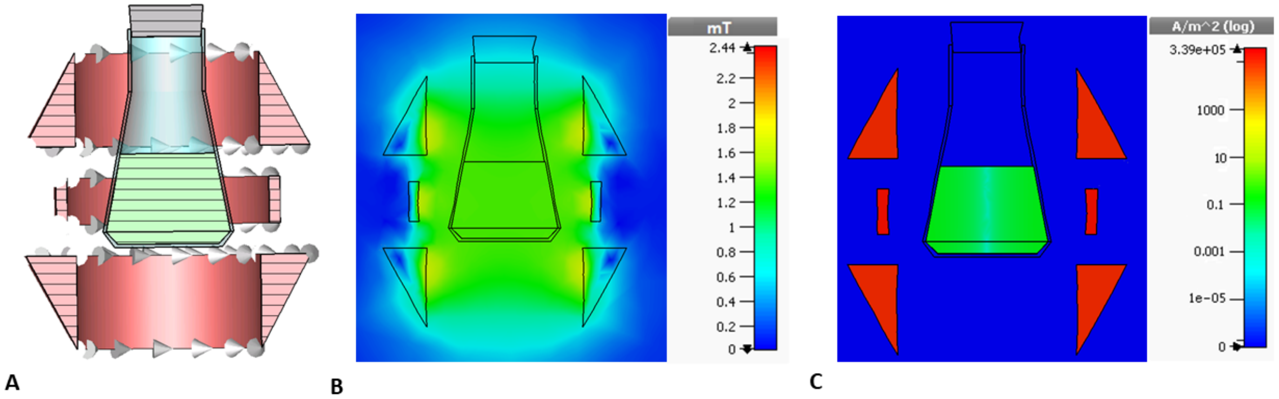

Figure S3. Exposure coil with cell solution in Erlenmeyer flask. **A.** Coil model. **B.** Distribution of magnetic flux density. **C.** Distribution of current density.
